# Numerical–Experimental Analysis toward the Strain Rate Sensitivity of 3D-Printed Nylon Reinforced by Short Carbon Fiber

**DOI:** 10.3390/ma15248722

**Published:** 2022-12-07

**Authors:** Hamid Reza Vanaei, Anouar El Magri, Mohammad Ali Rastak, Saeedeh Vanaei, Sébastien Vaudreuil, Abbas Tcharkhtchi

**Affiliations:** 1 Léonard de Vinci Pôle Universitaire, Research Center, 92916 Paris La Défense, France; 2 Arts et Métiers Institute of Technology, CNAM, LIFSE, HESAM University, 75013 Paris, France; 3 Euromed Research Center, Euromed Polytechnic School, Euromed University of Fes, Route de Meknès (Rond Point Bensouda), Fès 30 000, Morocco; 4 Department of Mechanical, Industrial and Aerospace Engineering, Concordia University, Montreal, QC H3G 1M8, Canada; 5 Dynamic and Smart System Laboratory, Department of Mechanical, Industrial and Manufacturing Engineering, University of Toledo, Toledo, OH 43606, USA; 6 Arts et Métiers Institute of Technology, CNRS, CNAM, PIMM, HESAM University, 75013 Paris, France

**Keywords:** 3D printing, mechanical behavior, strain-rate sensitivity, Nylon-CF

## Abstract

Despite the application of the Additive Manufacturing process and the ability of parts’ construction directly from a 3D model, particular attention should be taken into account to improve their mechanical characteristics. In this paper, we present the effect of individual process variables and the strain-rate sensitivity of Onyx (Nylon mixed with chopped carbon fiber) manufactured by Fused Filament Fabrication (FFF), using both experimental and simulation manners. The main objective of this paper is to present the effect of the selected printing parameters (print speed and platform temperature) and the sensitivity of the 3D-printed specimen to the strain rate during tensile behavior. A strong variation of tensile behavior for each set of conditions has been observed during the quasi-static tensile test. The variation of 40 °C in the platform temperature results in a 10% and 11% increase in Young’s modulus and tensile strength, and 8% decrease in the failure strain, respectively. The variation of 20 mm·s^−1^ in print speed results in a 14% increase in the tensile strength and 11% decrease in the failure strain. The individual effect of process variables is inevitable and affects the mechanical behavior of the 3D-printed composite, as observed from the SEM micrographs (ductile to brittle fracture). The best condition according to their tensile behavior was chosen to investigate the strain rate sensitivity of the printed specimens both experimentally and using Finite Element (FE) simulations. As observed, the strain rate clearly affects the failure mechanism and the predicted behavior using the FE simulation. Increase in the elongation speed from 1 mm·min^−1^ to 100 mm·min^−1^, results in a considerable increase in Young’s modulus. SEM micrographs demonstrated that although the mechanical behavior of the material varied by increasing the strain rate, the failure mechanism altered from ductile to brittle failure.

## 1. Introduction

Additive manufacturing (AM), also known as 3D printing, is a construction technology for the layer-by-layer production of a three-dimensional (3D) structure directly from a model [1,2]. The 3D printing technology has recently attracted great interest due to its low cost and faster construction [3]. A thermoplastic filament is extruded through a liquefier that can be heated to melt the filaments for layer-by-layer deposition on X-Y planes toward the Z-axis. Several types of thermoplastics, either polymers or composites, are increasingly used in this technique for this purpose. Regardless of the materials used in the 3D printing process, many process factors and physical phenomena play a crucial role in producing high-quality products. In fact, each of the mentioned characteristics directly affects the mechanical properties and strength of the final parts, which is related to the adhesion between the deposited layers [4,5,6,7,8]. In addition, efforts have been made to conduct research on a variety of materials, including acrylonitrile butadiene styrene (ABS), nylon, polycarbonate (PC), polylactic acid (PLA), and their related polymeric-based composites, in order to estimate their mechanical properties as a function of the aforementioned characteristics.

Hence, there are several studies on the effect of processing parameters on the quality of the final product. The tensile strength of 3D-printed polycarbonate parts was 75% more than that of extruded parts, according to a study evaluating their mechanical properties [9]. Furthermore, there are several papers that considered the mechanical behavior of the 3D-printed composites by investigating the effect of the particle or fiber reinforcements [10,11,12,13,14]. Zhong et al. [15] investigated the mechanical properties of 3D-printed glass-fiber-reinforced ABS polymer and they observed that the existence of fibers improved the inter-layer bonding between the deposited layers. In another study, Ning et al. [16] considered the 3D-printed carbon fiber-reinforced ABS polymer and found that the effect of carbon fiber was inevitable and increased the mechanical properties of the printed specimens. Melenka et al. [17] investigated the effect of continuous fiber-reinforced Nylon material on the mechanical strength of the fabricated parts, and they found an enhancement of the ultimate tensile strength.

Accordingly, using a new design gives us the opportunity to reach the optimized conditions. Therefore, it is important to apply the design approaches and engage the effect of individual variables (process parameters). In the 1920s, a concept named Design of Experiments (DOE) was proposed [18]. This technique is able to consider all the independent factors simultaneously. It offers an effective methodology for experimental characterization to define the related conditions, and one of its main advantages is the ability to evaluate the interaction of several parameters [19]. There is a possibility of implementing multi-variable testing methods at the same time. Considering the experimental characterizations, the Taguchi method is an appropriate optimization technique. Indeed, the most important essential feature of employing the Taguchi method is the design of the experiment to be implemented for process optimization. To do this, it is necessary to apply the DOE’s techniques in such purposes. One of them could be referred to as the Response Surface Method (RSM); a method that investigates the engagement of several dependent or independent parameters on the responses, including the regression analysis [20]. Qattawi et al. [21] applied this technique to evaluate the influence of several parameters on the mechanical behavior of 3D-printed PLA specimens and to find how they affect the strength of the material. In another study, Lee et al. [22] evaluated the optimal elastic properties of the 3D-printed ABS materials to see the impact of the engaged parameters on the mentioned feature.

To conclude, there is still a lack of research on the development of 3D-printed composite materials. Recently, 3D printing of fiber-reinforced polymers has become a promising point for the fabrication of structural components that are applicable in different fields of study such as biomedical and automotive industries. The Markforged^®^ 3D printer has achieved favorable attention due to its specific design and its potential to fabricate composite parts with a higher strength than conventional FFF 3D printers (available: https://markforged.com/products/). In fact, the 3D printer is capable of printing parts with Kevlar, Glass fiber composite, or continuous Carbon fiber reinforced Nylon polymer. Despite the application of the applied material, it is required to perform a thorough investigation to be able to optimize and consequently ameliorate the mechanical characteristics for obtaining high-quality final parts. The objective of this paper is to quantify and analyze the strain rate sensitivity of the 3D-printed tensile specimens. It is worth mentioning that the objective of the current paper is not to optimize the process; however, the focus on the strain rate sensitivity of the 3D-printed parts will help apply the obtained results for optimization purposes. At the outset, the nature of the mechanical strength of 3D-printed specimens and the individual effect of process variables is discussed using quasi-static tensile test. Then, their strain-rate sensitivity is analyzed by considering the best condition of printing to finally analyze their characteristics using both experimental tests and Finite Element simulations.

## 2. Materials and Methods

### 2.1. Material

A commercially available Onyx filament with a diameter of 1.75 mm has been analyzed. Some physico-chemical properties are presented in Table 1.

### 2.2. 3D Printer Device

A VOLUMIC 3D STREAM-Mk2 printer was used for 3D printing the Onyx specimens. This FFF system, equipped with both a heated bed and build chamber, has a build volume of 300 × 200 × 200 mm^3^. Tensile test specimens were printed directly on the heated glass plate. The infill parameter was set to 100% to obtain solid-like specimens. The major evaluated printing parameters are summarized in Table 2. All specimens were printed flat on the build platform (XY surface). Control of the printing parameters and slicing into individual layers was performed using SIMPLIFY 3D software (Version 5.0). From preliminary observations of printing specimens, it has been noticed that in certain values of the process parameters (as shown in Table 2), the quality of them is not acceptable. Therefore, based on this preliminary study, we have found a proper range for the parameters that the quality of the printed specimens is acceptable and thus, we have considered different values for platform temperature and print speed to define the printing conditions.

### 2.3. Characterization Methods and Experimental Procedure

#### 2.3.1. Microscopic Observation

A Quanta 200 ESEM (Thermo FEI, Eindhoven, The Netherlands) was employed for these SEM observations. It was configured with an EDAW (TSL) energy-dispersive spectroscopy and electron backscatter diffraction (EBSD) system to allow phase identification. In order to prevent surface charging, all analyzed specimens were first cryogenically fractured in liquid nitrogen, and then coated with a layer of electrically conducting gold (Au). A 15 kV acceleration voltage was used in a high-vacuum model.

#### 2.3.2. Mechanical Testing (Quasi-Static Tensile Test)

Tensile tests until failure have been carried out following ASTM D638 type IV at room temperature on INSTRON 4301 machine equipped with a 10 KN load cell and self-tightening jaws were used for each series of specimens.

**Figure 1:**
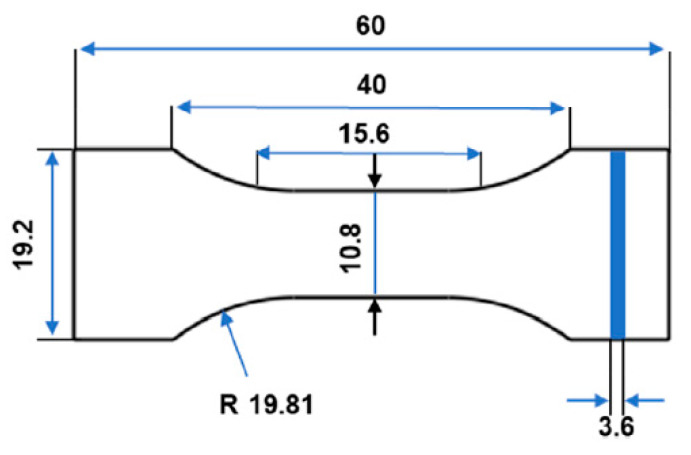
Printed specimens based on ASTM D638 type IV.

The printed specimen (based on ASTM D638 type IV) underwent a tensile test at 1, 10, 50, and 100 mm·min^−1^ (Figure 1). The selected elongation speeds were based on the fact that they cover a wide range of the strain rates for polymers and composites according to their application. Based on the tensile standard (ASTM D638 type IV), it has been suggested to consider 10 or 100 mm·min^−1^ the elongation speed as the most common elongation speed in industrial applications of such materials. Using a camera, a contactless approach is employed to measure the local deformation. The MTS Test Suite software collected displacement (mm) and force (N) and processed them to establish tensile strain-stress curves and determine tensile characteristics (Young’s modulus, tensile strength, and strain at break). The slope of the generated stress–strain curves were used to compute Young’s modulus. The tensile strength was determined by dividing the maximum applied force by the specimen’s cross-sectional area. Using a non-contact extensometer, the strain at failure was measured. All the provided values were derived as the average of at least five specimens.

### 2.4. Condition of Printing

The influence of platform temperature and print speed was taken into account by defining three conditions as displayed in Table 3. In fact, the defined conditions were chosen based on the previous paper and the obtained results in order to be able to define a suitable range of process variables. Five specimens were tested for each condition of tensile tests. After preliminary tests and according to the observations from the printed specimens, a range of 70–110 °C for platform temperature and 20–60 mm·s^−1^ for print speed was selected for this paper based on the printing flow and quality of trial parts. For proper printing, the liquefier temperature was fixed at 270 °C.

## 3. Results and Discussions

This study aims to investigate the influence of process parameters, namely platform temperature and print speed, on the mechanical properties of 3D-printed Nylon parts. Regarding the application of this material, efforts have been made to distinguish the optimal printing conditions based on its tensile behavior and then to apply them at various elongation speeds.

### 3.1. Individual Effect of Process Parameters on Mechanical Properties of 3D-Printed Onyx

**Figure 2:**
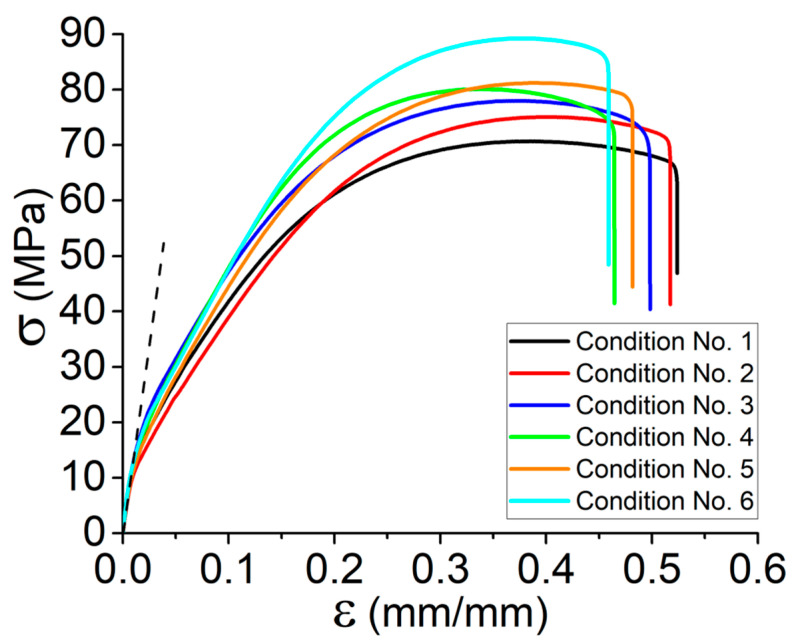
Tensile behavior for the set of specimens according to conditions 1–6 representing the variation of platform temperature and print speed.

Figure 2’s accompanying graphs depict the results of tensile tests conducted on the set of specimens examined in this research (all tests have been done at room temperature). In order to have a better understanding of the characteristics of the printed dog-bone specimens, at least five tensile tests were conducted for each condition. As can be seen, each defined condition results in distinct behaviors at varied platform temperatures and print speeds. The obtained data indicate that variations in both print speed and platform temperature increase the specimens’ ultimate strength. Nevertheless, their individual effects are different, and each parameter plays a unique function, as demonstrated by Table 4’s data. At constant values of liquefier and platform temperature, the effect of print speed is more significant. The specimens that were printed under condition No. 6 (T_platform_ = 110 °C and V = 60 mm·s^−1^) have the highest ultimate strength. The same condition exists for the variation of Young’s modulus as was seen for those printed under condition No. 6 with the highest value. Taking into account the lowest platform and print speed values (condition No. 1) as well as the highest ones (condition No. 6), Young’s modulus and ultimate strength rise by approximately 27% and 25%, respectively. In fact, adjusting the cooling rate and, subsequently, the temperature of the deposited layers during printing by changing the platform temperature and even the print speed would improve the adhesion of the material. Temperature control enables the material to be hot enough in the targeted zone for greater material penetration and, consequently, better adhesion [23]. Despite the heat transfer across the deposited layers, it seems that these differences could not be avoided as platform temperature has its own effect

Furthermore, given the above-mentioned explanations and the presented curves in Figure 2 and data collected in Table 4, the overall results could be summarized as follows:

- By increasing the platform temperature from 70 °C to 110 °C in the same print speed, Young’s modulus and tensile strength increased by 10% and 11%, respectively.
- By increasing the print speed in the range of 40 mm·s^−1^ to 60 mm·s^−1^ (e.g., conditions No. 1 and No. 4), the tensile strength of the Onyx increased by 14%.
- By increasing the platform temperature from 70 °C to 110 °C for the same print speed (e.g., conditions No.1 and No. 3), the failure strain decreased by 8%.
- By increasing the print speed in the range of 40 mm·s^−1^ to 60 mm·s^−1^ (e.g., conditions No. 3 and No. 6), the failure strain decreased by 11%.

**Figure 3:**
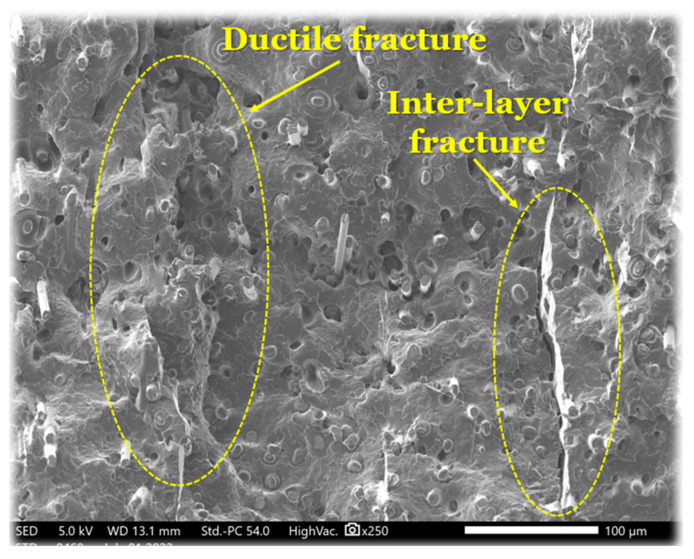
SEM micrograph for tensile fracture surface of the specimen in condition No. 6.

Figure 3 is a SEM micrograph of the fracture surface of a specimen under condition No. 6. On the basis of the preceding explanations on the tensile behavior of printed specimens under various conditions, it has been seen that the material’s ductility has decreased and that the cracked surface indicates a more brittle failure. Nonetheless, there are still zones of ductile failure that show the failure of the matrix compared to the reinforced fibers, as briefly described in the 3D printing method. By increasing the platform temperature and also the print speed, the inter-penetration of materials would be facilitated, and depending on the material’s morphology as an amorphous or semi-crystalline material, the critical zones of possible ductile fracture would be created. However, the inter-layer fracture distance from the ductile fracture may be associated with the printing direction (Z direction). The higher distance from the platform results in an inhomogeneity due to the variation of the temperature gradient, giving in a variable solidification rate [8]. Another remarkable remark according to the SEM images (Figure 3) refers to the orientation of the carbon fibers that are perpendicular to the cross-section of the fractured surface. It is also noticeable that there is a poor adhesion between the fiber and matrix as can be observed by the holes that are visible accordingly. Plastic deformation of the matrix is also noticeable as it determines the ductility of the 3D-printed material. 

### 3.2. Tensile Properties as a Function of Strain Rate

**Figure 4:**
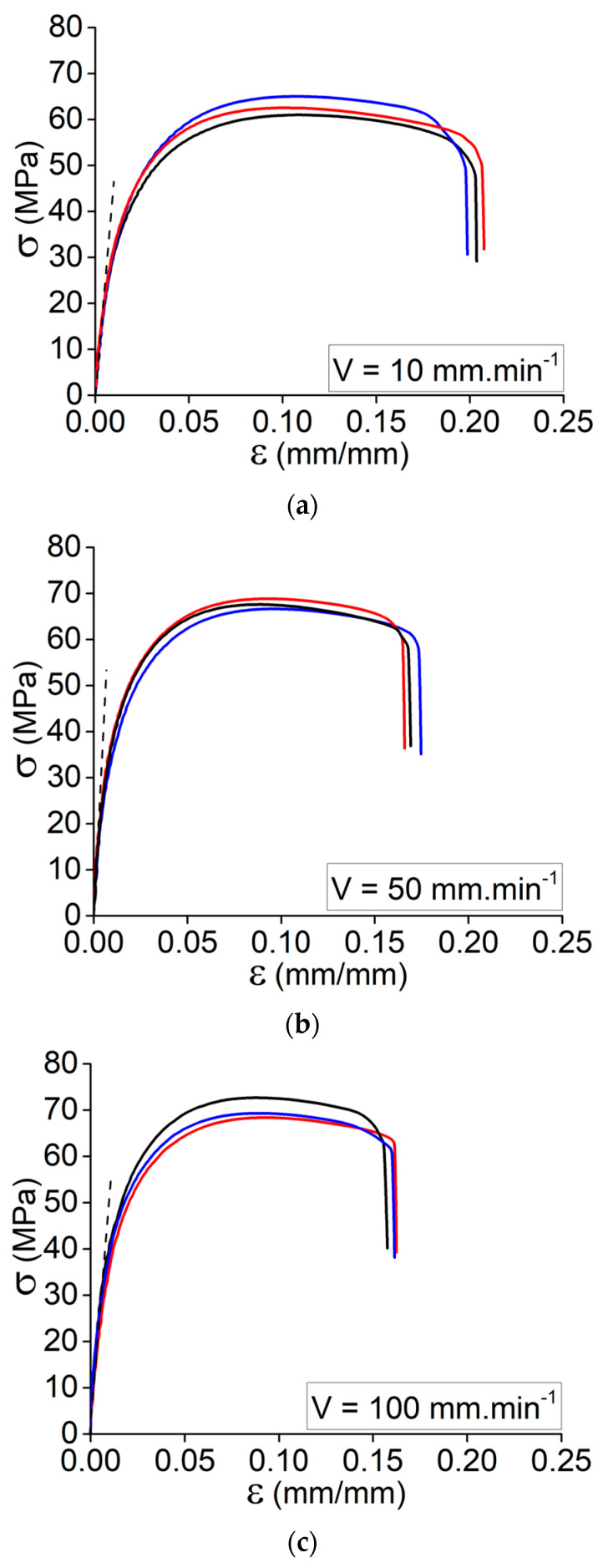
Tensile behavior for the set of specimens according to condition No. 6 at different elongation speed: (**a**) V = 10 mm·min^−1^, (**b**) V = 50 mm·min^−1^, and (**c**) V = 100 mm·min^−1^.

In this study, the effect of strain rate on the tensile strength and Young’s modulus of Onyx composite at different elongation speeds V = 10, 50, and 100 mm·min^−1^ is studied. The tests were conducted on the printed specimens based on the process variables of the best condition, No. 6. In fact, a comparison of the acquired tensile data for conditions No. 1 through No. 6 revealed that the printed specimens based on condition No. 6 exhibited a higher Young’s modulus and tensile strength. Worth mentioning that this is the situation with the highest print speed and platform temperature. In accordance with the previously stated tensile data, Onyx demonstrated an area of weak elasticity and no discernible plastic deformation. The outcomes clearly demonstrate the strain rate sensitivity of the Onyx specimens produced by 3D printing. As stated, Figure 4a–c depicts the experimental results obtained at various elongation speeds (three specimens per elongation speed). Presumably, the increase in elongation speed and, subsequently, the strain rate caused the material to exhibit a brittle characteristic.

Following the presented results in Figure 4, the data collected in Table 5 indicates the recorded values for Young’s modulus, ultimate stress, and the elongation of ultimate stress for the printed specimens according to condition No. 6 at different elongation speeds. The overall results could be taken into account as follows:

- By increasing the elongation speed from 1 mm·min^−1^ to 100 mm·min^−1^, there is a considerable increase in Young’s modulus.
- By increasing the elongation speed from 1 mm·min^−1^ to 10 mm·min^−1^, the ultimate stress decreases and then increases by increasing the elongation speed to 100 mm·min^−1^.
- The sudden reduction in the ultimate strength might be related to the variation of the failure mechanism from ductile failure to brittle failure (rapid matrix failure).
- By increasing the elongation speed from 10 mm·min^−1^ to 100 mm·min^−1^, the ultimate stress increases. It is still lower than the first elongation speed (failure mechanism: ductile to brittle failure).
- By increasing the elongation speed from 1 mm·min^−1^ to 10 mm·min^−1^, the strain of ultimate stress decreases, whereas it increases by further enhancement of the elongation speed.

**Figure 5:**
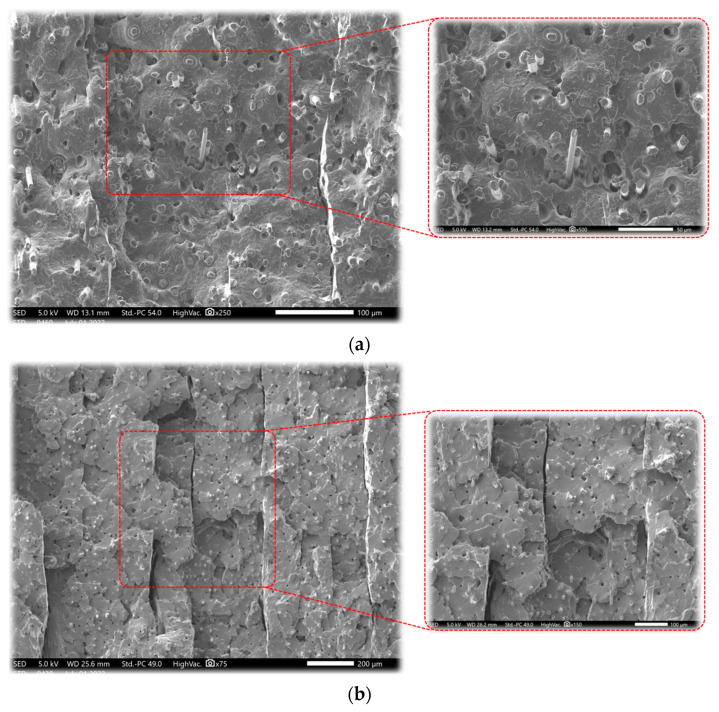
SEM micrograph for tensile fracture surface of the specimen in condition No. 6 for (**a**) V = 1 mm·min^−1^, (**b**) V = 100 mm·min^−1^.

Accordingly, to have a better representation of the above-explained issues, SEM micrographs of a failed specimen at an elongation speed of 1 mm·min^−1^ and 100 mm·min^−1^ are shown in Figure 5. They clearly show the failure mechanism that tends to change from ductile failure to brittle failure by increasing the elongation speed. As shown in Figure 5a, there is a ductile failure toward the fracture surface and no sign of inter-layer failure. It is worth mentioning that due to the mechanism of layer-by-layer deposition in the 3D printing process, failure is disposed to occur at the layers’ interfaces. However, at the elongation speed of 100 mm·min^−1^ (Figure 5b), higher elongation speed produces a brittle failure, particularly at the interface of the deposited layers. This point could be related to the fact that at a certain value of strain rate (or elongation speed), matrix failure would happen more quickly, and thus the reinforcement fibers would take action in the failure mechanism as well [6]. As mentioned in the previous section, the fiber orientation is mainly perpendicular to the fracture surface according to Figure 5 for different strain rates. Furthermore, it is also noticeable to mention that more voids on the failure cross-section could be observed by increasing the strain rate.

It is possible to use the m-index to determine the effect of strain rate on the mechanical behavior of 3D-printed specimens. It is defined as the slope of the Lnσ- Lnε curve (m = Lnσ/Lnε) with the associated elongation speed. Table 6 illustrates the periodical variation of the m-index parameter as well as the unique influence of each elongation speed. Nonetheless, it is essential to state the threshold for the index that divides the super-elasticity and super-plasticity regions in terms of the manufactured specimens.

### 3.3. Simulation Validation

**Figure 6:**
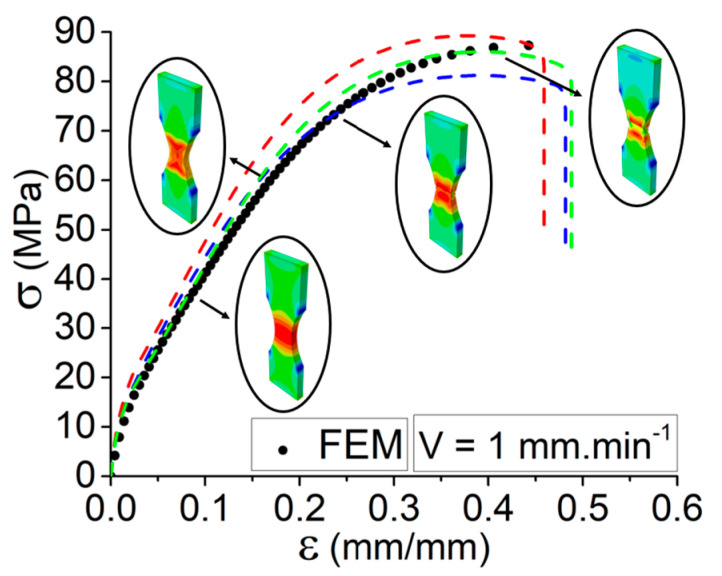
Comparison through the simulated and experimental tensile behavior for the specimens at elongation speed of V = 1 mm·min^−1^.

Finite Element Modeling (FEM) is found to be able to evaluate and analyze the structural behavior of the 3D-printed composites, including carbon/fiber deposited layer by layer in the desired geometry [24]. In order to study the mechanical behavior of specimens, a 3D model was modeled in ABAQUS [25]. In this regard, Finite Element calculations were implemented to validate and check the tensile behavior of the 3D-printed specimen at different elongation speeds. The material was assumed as isotropic with the properties as defined in the Abaqus modules for the prediction of the mechanical behavior through tensile tests. In this regard, the equivalent properties are considered for the modeling. The bottom surface of the model, similar to the actual experimental tests, is fixed, and the top surface of the model undergoes incremental Forces. As indicated in Figure 6, the tensile behavior of the simulated condition represents good agreement with a set of tensile curves for the elongation speed of 1 mm·min^−1^. In fact, the elastic region of the simulated curve predicts a good accuracy with the mechanical behavior of the set of specimens that are subjected to the tensile test. The FEM results indicate that the shape and tensile behavior of the models are in conjunction with the elongation speed. More interestingly, the same conclusion can be made from experimental tensile behaviors. 

Moreover, there is a slight difference in the plastic region. One of the explanations for such a slight difference is related to the condition of printing and manufacturing of the specimens. In fact, none of the specimens is ideal and they are likely vulnerable to have some deviation during the printing process. In the case of defining the plastic behavior of material to the software, the plastic region is more complicated compared to the linear region.

Accordingly, different elongation speeds were implemented in the FE simulation to predict the tensile behavior of the 3D-printed specimens at different strain rates. It is important to validate the experimental tests that have been implemented for better characterization of the characteristics of the material for further investigations. FE simulation for the tensile behavior of the 3D-printed specimens was repeated with the same conditions in different elongation speeds (V = 10, 50, and 100 mm·min^−1^)

**Figure 7:**
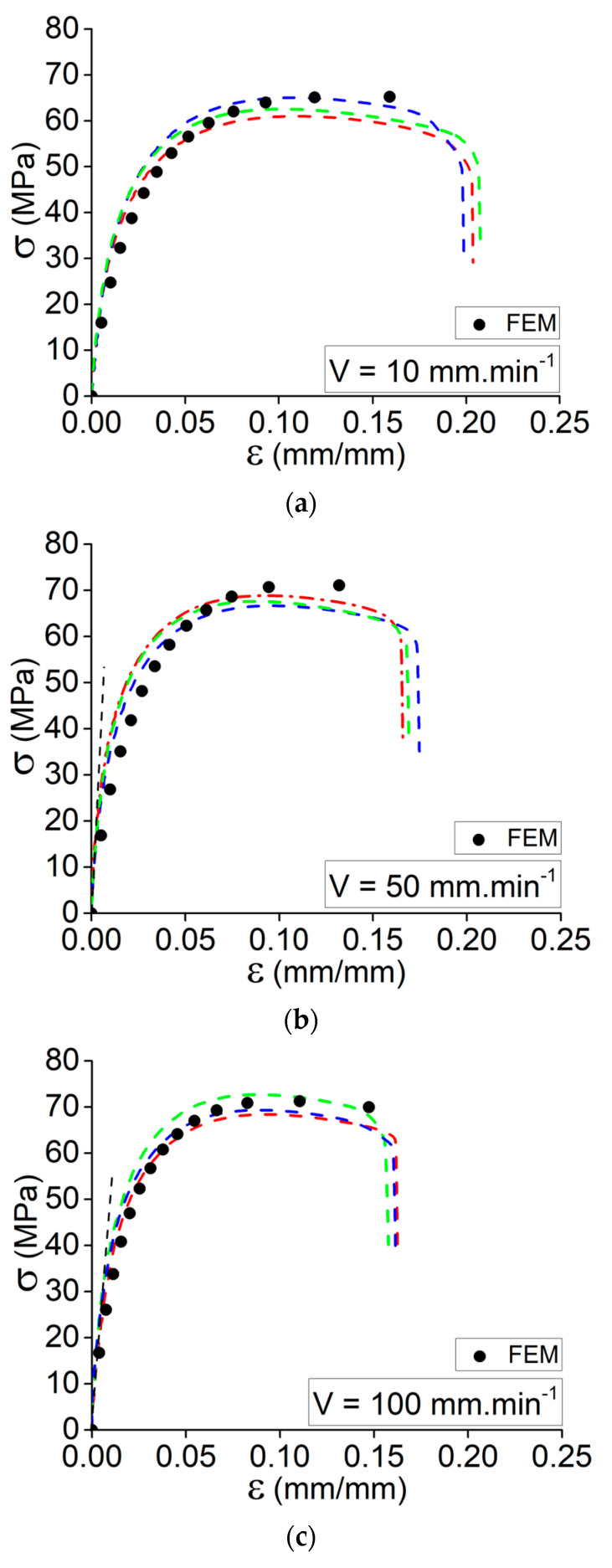
Comparison through the simulated and experimental tensile behavior for the specimens at elongation speeds of (**a**) V = 10 mm·min^−1^, (**b**) V = 50 mm·min^−1^, and (**c**) V = 100 mm·min^−1^.

Figure 7a–c demonstrates that, despite the good accuracy of the experimental testing at different elongation speeds, there are some variations in the plastic regime of the tensile curves. Due to the layer-by-layer deposition mechanism of 3D printing, the interaction of multiple parameters may have a significant impact on the adhesion between the deposited layers. Hence, it is essential to consider this issue while characterizing such studies. From the accompanying graphs in Figure 7a–c, it is noticeable that the slope of the curve (Young’s modulus variation) has been accurately predicted for the three elongation rates in the elastic regime, confirming the observed behavior in the assessed study so far. As there are some deviations from the experimental curves in the plastic regime, there is a potential for self-heating in the specimen due to the increase in strain rate applied to the specimen. It could be a source of changing the failure mechanism from ductile to brittle failure as the matrix faces temperature variations. This issue has been thoroughly explained in the authors’ prior paper regarding how cyclic stresses can increase the specimen’s self-heating during the test [26].

## 4. Conclusions

The mechanical behavior of 3D-printed Onyx specimens under quasi-static loadings is influenced mainly by several parameters such as those related to the source of temperature during the parts’ construction as well as layer thickness and manufacturing orientation.

In this research paper, the mechanical behavior of Onyx specimens was investigated, including the individual effect of process parameters such as print speed and platform temperature, further with the strain rate sensitivity of the printed specimens (liquefier temperature was fixed at T_Liq_ = 270 °C). The following results have been summarized:

- A strong variation of tensile behavior for each set of conditions has been observed during the quasi-static tensile test.
- The variation of 40 °C in the platform temperature results in a 10% and 11% increase in Young’s modulus and tensile strength and an 8% decrease in the failure strain, respectively.
- The variation of 20 mm·s^−1^ in print speed results in a 14% increase in the tensile strength and an 11% decrease in the failure strain.
- The individual effect of process variables is inevitable and affects the mechanical behavior of the 3D-printed composite, as observed from the SEM micrographs (ductile to brittle fracture).
- Increase in the elongation speed from 1 mm·min^−1^ to 100 mm·min^−1^, results in a considerable increase inYoung’s modulus.
- The sudden reduction in the ultimate strength might be related to the variation of the failure mechanism from ductile failure to brittle failure (rapid matrix failure).
- SEM micrographs demonstrated that although the mechanical behavior of the material varied by increasing the strain rate, the failure mechanism altered from ductile to brittle failure.

## Figures and Tables

**Table 1:** Characteristics of the applied Onyx filament (adapted from manufacturer’s datasheet).

Properties	Typical Value
Material Density	1.2 g·cm^−3^
Diameter (Tolerance)	1.75 mm (±0.01 mm)

**Table 2:** Process variables of the 3D-printing process.

Printing Parameters	Value
Liquefier temperature (°C)	270
Platform temperature (°C)	70–90–110
Chamber temperature (°C)	30
Print speed (mm/s)	40–60
Layer height (mm)	0.15
Infill density (%)	100
Infill pattern	line
Number of bottom/top layers	2/2
Number of contours (wall)	2
Infill line directions ( relative to the long axis of the test bar) (°)	(45/−45)

**Table 3:** Various conditions of printing.

Condition	Liquefier Temperature (°C)	Platform Temperature (°C)	Print Speed (mm·s^−1^)
1	270	70	40
2		90	
3		110	
4		70	60
5		90	
6		110	

**Table 4:** Results of tensile behavior of printed specimens according to conditions No. 1–6.

Conditions	E (GPa)	$\sigma_{max}\;(\mathrm{MPa})$	$\varepsilon \;at\;\sigma_{max}\;(\mathrm{mm}\cdot \mathrm{mm}{-}^{1})$
1	1.5 ± 0.12	70.7 ± 1.3	0.39 ± 0.021
2	1.4 ± 0.09	75 ± 1.0	0.39 ± 0.020
3	1.7 ± 0.13	78 ± 1.2	0.38 ± 0.023
4	1.8 ± 0.10	80.1 ± 1.2	0.34 ± 0.022
5	1.8 ± 0.11	81.2 ± 1.1	0.39 ± 0.022
6	1.9 ± 0.11	89.3 ± 1.2	0.37 ± 0.021

**Table 5:** Results of tensile behavior of printed specimens according to conditions No. 6 for different elongation speeds.

Elongation Speed (mm·min^−1^)	E (GPa)	$\sigma_{max}\;(\mathrm{MPa})$	$\varepsilon \;at\;\sigma_{max}\;(\mathrm{mm}\cdot \mathrm{mm}{-}^{1})$
1	1.9 ± 0.12	89.3 ± 1.2	0.37 ± 0.021
10	3.5 ± 0.10	63 ± 1.1	0.1 ± 0.022
50	4.6 ± 0.11	67 ± 1.0	0.95 ± 0.020
100	5.1 ± 0.12	71 ± 1.3	0.89 ± 0.021

**Table 6:** Results of tensile behavior of printed specimens according to conditions No. 6 for different elongation speeds.

Elongation Speed	1 mm·min^−1^	10 mm·min^−1^	50 mm·min^−1^	100 mm·min^−1^
m-index	0.564	0.376	0.407	0.321

## Data Availability

Not applicable.
